# Magnitude and determinants of neural tube defect in Africa: a systematic review and meta-analysis

**DOI:** 10.1186/s12884-021-03848-9

**Published:** 2021-06-14

**Authors:** Daniel Atlaw, Yohannes Tekalegn, Biniyam Sahiledengle, Kenbon Seyoum, Damtew Solomon, Habtamu Gezahegn, Zerihun Tariku, Yared Tekle, Vijay Kumar Chattu

**Affiliations:** 1 Department of Human Anatomy, School of Medicine, Goba Referral Hospital, Madda Walabu University, Goba, Ethiopia; 2 Department of Public Health, School of Health Science, Goba Referral Hospital, Madda Walabu University, Goba, Ethiopia; 3 Department of Midwifery, School of Health Science, Goba Referral Hospital, Madda Walabu University, Goba, Ethiopia; 4 Department of physiology, School of Medicine, Goba Referral Hospital, Madda Walabu University, Goba, Ethiopia; 5 College of Medicine and Health Sciences, Dire Dawa University, Dire Dawa, Ethiopia; 6 Department of Medicine, Faculty of Medicine, University of Toronto, Toronto, ON M5S 1A8 Canada

**Keywords:** Anencephaly, Spina bifida, Neural tube defect, Systematic review, Africa

## Abstract

**Background:**

Neural tube defects (NTDs) are a group of disorders that arise from the failure of the neural tube close between 21 and 28 days after conception. About 90% of neural tube defects and 95% of death due to these defects occurs in low-income countries. Since these NTDs cause considerable morbidity and mortality, this study aimed to determine the prevalence and associated factors of NTDs in Africa.

**Methods:**

The protocol of this study was registered in the International Prospective Register of Systematic Reviews (PROSPERO number: CRD42020149356**).** All major databases such as PubMed/MEDLINE, EMBASE, CINAHL, Web of Science, African Journals Online (AJOL), and Google Scholar search engine were systematically searched. A random-effect model was used to estimate the pooled prevalence of NTDs in Africa, and Cochran’s Q-statistics and I^2^ tests were used to assess heterogeneity between included studies. Publication bias was assessed using Begg ’s tests, and the association between determinant factors and NTDs was estimated using a random-effect model.

**Results:**

Of the total 2679 articles, 37 articles fulfilled the inclusion criteria and were included in this systematic review and meta-analysis. The pooled prevalence of NTDs in Africa was 50.71 per 10,000 births (95% CI: 48.03, 53.44). Folic acid supplementation (AOR: 0.40; 95% CI: 0.19–0.85), maternal exposure to pesticide (AOR: 3.29; 95% CI: 1.04–10.39), mothers with a previous history of stillbirth (AOR: 3.35, 95% CI: 1.99–5.65) and maternal exposure to x-ray radiation (AOR 2.34; 95% CI: 1.27–4.31) were found to be determinants of NTDs.

**Conclusions:**

The pooled prevalence of NTDs in Africa was found to be high. Maternal exposure to pesticides and x-ray radiation were significantly associated with NTDs. Folic acid supplementation before and within the first month of pregnancy was found to be a protective factor for NTDs.

**Supplementary Information:**

The online version contains supplementary material available at 10.1186/s12884-021-03848-9.

## Background

A neural tube defect (NTD) is a failure of the neural tube to close during the 3rd and 4th weeks of pregnancy [1]. The development and closure of the neural tube are typically completed within 28 days after conception before many women are aware that they are pregnant [2]. Neural tube defects can be identified through simple prenatal testing using ultrasound imaging or maternal serum alpha-fetoprotein level screening [3].

Neural tube defect is estimated to affect about 300,000 newborns worldwide in 2016 [4], resulting in about 88,000 deaths per year [4]. In low-income countries, NTDs may account for 29% of neonatal deaths due to observable birth defects [4]. The burden of NTDs in developing countries have been reported to be two times higher than in developed countries [5]. In Africa, the median NTD prevalence was 11.7 per 10,000 births [4]. A review conducted in 2016 stated that nearly 270,000 NTDs occur due to lack of folic acid fortification; this estimate, however, might be impacted due to the paucity of data [6].

From different forms of neural tube defects, anencephaly and spina bifida are prevalent in developing countries [7]. Anencephaly is invariably associated with death as a stillbirth, a neonatal death, and a post-neonatal death [8]. In many cases, NTDs will end up in different forms of complications like mortality, disabilities, and psychological disorders of affected families [8].

Neural tube defects do not have specific causative agents, but genetic, environmental, and maternal age factors were among common contributors [9]. Maternal hypertension and maternal fever during pregnancy were identified as risk factors for NTDs [10]. Maternal history of alcohol intake during pregnancy was found to be significantly associated with NTDs [11]. Consanguineous marriage is also listed as a common factor for NTDs [12].

Neural tube defects are among commonly avoidable defects; it is estimated that 50–60% of these defects could be prevented by achieving and maintaining adequate folate levels before and within the first month of pregnancy [13]. A global review conducted in 2014 tried subgroup analysis for Africa on the burden of neural tube defect. Still, they have included limited studies which may affect the overall magnitude of NTDs [3]. Even though some global reviews have been conducted to assess the burden of NTDs, none of them reported determinant factors [3, 4, 6, 8]. Therefore, this systematic review and meta-analysis aimed to determine the pooled prevalence and determinants of NTDs in Africa. Determining the most avoidable risk factors will assist policymakers in designing strategies to decrease the burden of neural tube defects in Africa.

## Methods

### Study protocol

The protocol of this study was registered in the International Prospective Register of Systematic Reviews (PROSPERO), the University of York Centre for Reviews and Dissemination (ID number: CRD42020149356) [14]. This review and meta-analysis were conducted according to the guideline of Preferred Reporting Items for Systematic reviews and Meta-Analysis (PRISMA) (additional file 1) [15].

### Search strategy

A systematic review and meta-analysis were conducted using published and unpublished articles on the prevalence and associated factors of NTDs in Africa. The databases used to search for studies were PubMed, EMBASE, Google Scholar, CINAHL, POPLINE, and African Journals Online (AJOL), and grey literature was searched on Google and Research Gate. The following vital terms neural AND tube AND defect AND “determinant factors” OR “associated factors” OR “protective factors” OR “risk factors” AND “Africa countries” were used separately or in combination with the Boolean operator’s terms “AND” and “OR.” The search was also done by combining the above search terms with the names of all countries included in Africa and the sub-region of Africa (additional file 2). The reference lists of the retrieved studies were also scanned to access additional articles and screened against our eligibility criteria.

### Eligibility criteria

Any study in Africa that reported magnitude and determinant factors for NTDs and fulfilled the following criteria were recruited into the analysis:

#### Study area

All studies conducted in African countries.

#### Population

Epidemiological studies had reported prevalence and risk factors of NTDs as an outcome.

#### Study designs

All observational studies (cross-sectional, case-control, and cohort) reporting the prevalence and determinants of NTDs were eligible for this systematic review and meta-analysis.

#### Language

Articles published in English were considered.

#### Publication status

Both published and unpublished articles were considered.

#### Study period

No restriction of the period applied to this review.

### Study selection

Important articles identified from the databases mentioned above and websites were imported into an Endnote X8, and duplicates were removed. Screening retrieved articles titles, abstracts, and full-text quality was conducted independently by two review authors (DA & KS) based on the eligibility criteria. The disagreement between the two reviewers was resolved by reaching a consensus through discussion.

### Risk of bias assessment

The study risk of bias was assessed using the Joana bridge institute (JBI) critical appraisal tool [16]. Two authors (DA & KS) evaluated the quality of the full text considered to be included in the meta-analysis. The tool consists of ten items for case-control and eight for cross-sectional studies (additional files 3 and 4). Each item for each study was judged as Yes (1) and No (0). When the information provided was not adequate to make a judgment for a specific item, we agreed to grade that item with a ‘No’ (0). Each study was graded depending on the number of items judged ‘Yes’ (1) as low risk (≥ 7), medium risk (5 to 6) or high risk (≤ 4) for cross-sectional studies and low risk (≥8), medium risk (7 to 6) and high risk (< 5) for case-control studies (additional files 3 and 4).

### Data extraction

The selected papers were thoroughly reviewed, and the required information for the systematic review was extracted and summarized using an extraction table in Microsoft Office Excel software (additional files 5 and 6).

The data extraction tool consists of the name of the author (s), country and sub-region, study design, setting, year of publication, sample size, and number of NTDs (additional file 5). Data were extracted in two-by-two tables for determinants of NTDs, pooled odds ratio with their corresponding 95% confidence interval (CI) was calculated based on the original studies report (additional file 6).

### Statistical methods and analysis

The extracted data were imported into STATA/SE version 14 software for all statistical analysis. The heterogeneity among all included studies was assessed by using the I^2^ statistics and Cochran Q test. In this meta-analysis, the tests indicate significant heterogeneity among included studies (I^2^ = 100, *P*-value <.001). For this reason, we used a random-effects model as a method of analysis. The publication bias was assessed using begg ‘s test statistics. Pooled prevalence and odds ratios along their corresponding 95% CI were presented using a forest plot. Subgroup analyses for the prevalence of NTDs were performed by sub-regions of Africa. To determine factors associated with NTDs, data were entered into Review Manager Version 5, and pooled odds ratios (ORs) with 95% confidence interval (CI) were used.

### Operational definition

Neural tube defects all newborns having one of the following defects are considered as having NTD. Spina bifida (meningocele and myelomeningocele), anencephaly, and encephalocele.

Prevalence of NTDs = number of a newborn with NTDs/total number of newborns *100

## Results

The findings from this systematic review and meta-analysis are described in various sub-headings described below.

### Description of included studies

**Fig. 1:**
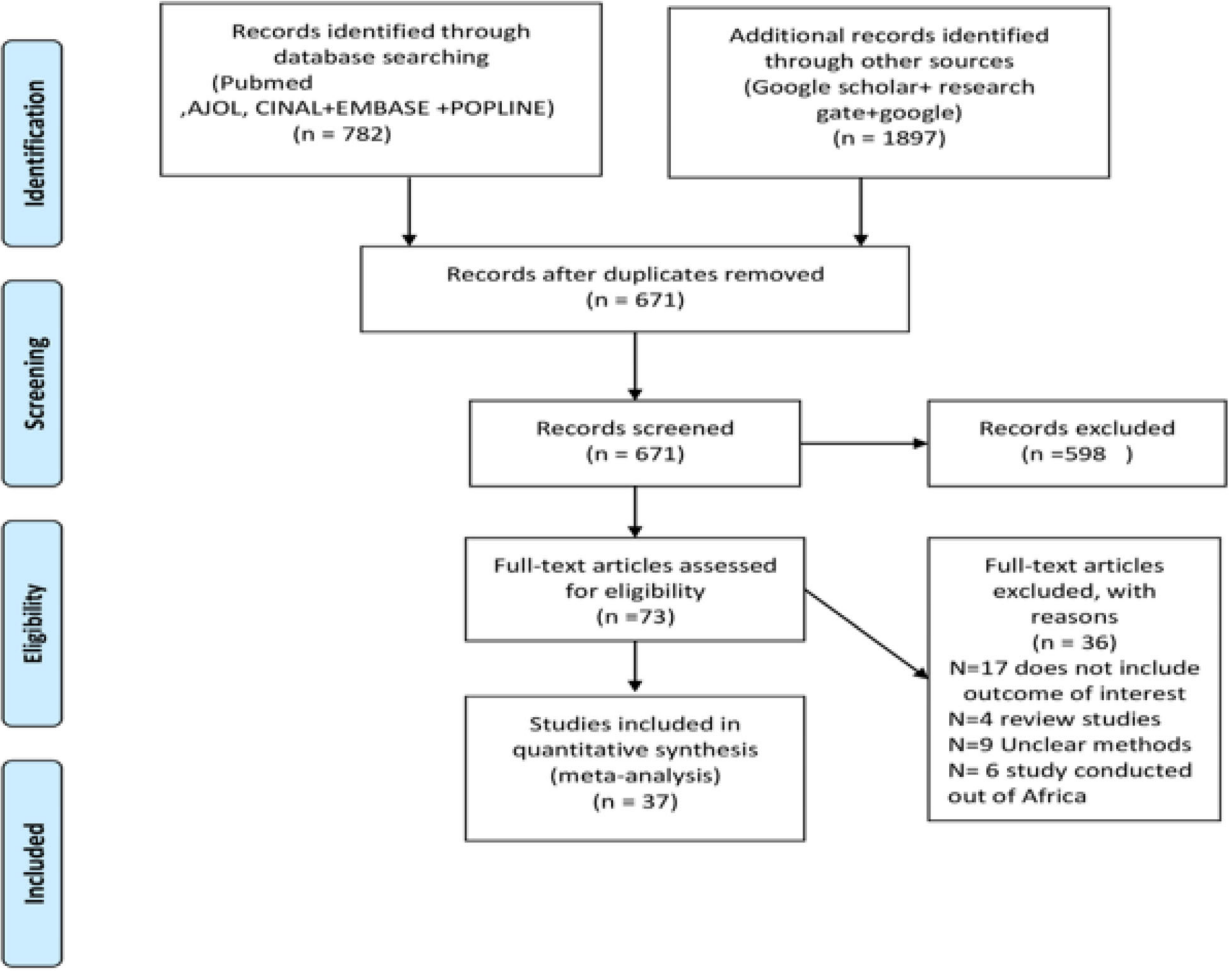
PRISMA flow diagram, for systematic review on prevalence and factors associated with neural tube defects in Africa

A total of 2679 published articles were retrieved through the search strategies. Then, 2008 records were removed due to duplication. Furthermore, 598 records were excluded after screening by title and abstract. Finally, a total of 73 full-text articles were screened against the eligibility criterion, of which 37 articles meet the requirements to be included in the final analysis (Fig. 1).

### Characteristics of the included studies

Fourteen African countries were represented in this review. Of these, 9 (24.3%) of the studies were from West African [17–25], 18 (48.6%) were from East African countries [26–43], 1(2.7%) from Central Africa [44], 1 (2.7%) from Southern Africa [45], and 8 (21.6%) were from Northern African country [46–53]. Regarding the study design, twenty-nine (78%) were cross-sectional, and eight (22%) were case-control studies. Studies were categorized according to their quality; ten studies were considered to have high quality 10 (27%), twenty-six medium quality 26(70%), and one study as having high low quality (Tables 1 and 2).

**Table 1:** Characteristics of included studies for prevalence of neural tube defects in Africa

Authors	year	Country	Design	Setting	sample size	number of NTD	Prevalence per 10,000	SB per 10,000	Anecephaly per 10,000	Risk of bias
Abbey et al. [25]	2017	Nigeria	cross-sectional	Hospital based	7670	28	36.51	31.29	5.22	Medium
Genti et al. [29]	2015	Ethiopia	cross-sectional	Hospital based	45,951	156	33.95	11.09	15.23	Medium
Ahuka et al. [43]	2006	D.Congo	cross-sectional	Hospital based	8824	9	10.19	6.79	3.39	Medium
Alhassan et al. [19]	2017	Ghana	cross-sectional	Hospital based	35,426	57	16.09	5.93	0.85	Medium
Anyanwu [17]	2020	Nigeria	cross-sectional	Hospital based	1456	4	27.47			Medium
Alem et al. [26]	2018	Ethiopia	cross-sectional	Hospital based	14,903	195	130.84	64.42	66.43	Low
Buccimazza et al. [45]	1994	South Africa	cross-sectional	Hospital based	516,252	606	11.74	1.67	0.91	Medium
Mumpe-Mwanja et al. [42]	2019	Uganda	cross-sectional	Hospital based	69,766	72	10.32			Medium
Ekanem et al. [22]	2008	Nigeria	cross-sectional	Hospital based	127,929	110	8.59	3.67	1.56	Medium
Ekwochi et al. [21]	2017	Nigeria	cross-sectional	Hospital based	5830	6	10.29	6.86	3.43	Low
EL-Mogharabi et al. [12]	2019	Libiya	cross-sectional	Hospital based	16,765	18	10.74	5.37	5.37	Medium
Estifanos et al. [36]	2017	Ertrea	cross-sectional	Hospital based	39,803	185	46.48	6.03	17.34	Medium
Adane et al. [28]	2018	Ethiopia	cross-sectional	Hospital based	19,650	103	52.41	29.01	3.56	Medium
Haucher et al. [46]	2008	Algeria	cross-sectional	Hospital based	28,500	215	75.44	43.51	31.23	Medium
Aride et al. [20]	1992	Nigeria	cross-sectional	Hospital based	5799	42	72.43	55.18	17.24	Medium
Laamiri et al. [53]	2014	Morocco	cross-sectional	Hospital based	819,224	330	4.03	1.81	2.22	Medium
Nasri et al. [48]	2014	Tunisia	cross-sectional	Hospital based	3,803,889	769	2.02	0.79	1.23	Medium
Mekonen et al. [34]	2015	Ethiopia	cross-sectional	Hospital based	1516	20	131.93	125.33	6.59	Medium
Omar et al. [47]	2016	Sudan	cross-sectional	Hospital based	36,785	103	28.00	19.30	8.69	Low
Radouani et al. [48]	2015	Morocco	cross-sectional	Hospital based	60,017	80	13.33	5.17	8.16	High
Singh et al. [49]	2000	Libiya	cross-sectional	Hospital based	16,186	18	11.12	10.50	0.62	Medium
Sorri et al. [31]	2015	Ethiopia	cross-sectional	Hospital based	28,961	177	61.12	28.31	32.80	Medium
Taye et al. [33]	2019	Ethiopia	cross-sectional	Hospital based	76,201	612	80.31	127.95	27.29	Low
Eka et al. [23]	2016	Nigeria	cross-sectional	Hospital based	7329	72	98.24	66.86	13.64	Low
Gadafaw et al. [38]	2017	Ethiopia	cross-sectional	Hospital based	8677	111	127.93	51.86	76.06	Low
Mitiku et al. [27]	2017	Ethiopia	Cross-sectional	Hospital based	84	2	238.09		238.09	High
Legese et al. [30]	2019	Ethiopia	cross-sectional	Hospital based	876	6	68.49	22.83	45.66	Low
Nandi and Singh [24]	2018	Nigeria	cross-sectional	Hospital based	10,163	22	21.64	15.74338	5.90	Medium
Taye et al. [32]	2016	Ethiopia	cross-sectional	Hospital based	319,776	1010	31.58	10.19	1.13	Low

**Table 2:** Characteristics of included studies for determinants of neural tube defect in Africa

Authors /Year	Design	Country	Population	Total sample	Case	Control	Risk of bias	Result	OR 95%(CI)
Shabrawi et al. 2015 [52]	Case-control	Egypt	Newborn and mothers	180	62	118	Medium	folic acid supplementation	1.27(.62–2.62)
								paternal consanguineous marriage	3.5 (1.70–7.10)
Nasri et at 2015 [51]	Case-control	Tunisia	Newborn and mothers	132	64	68	Medium	maternal age greater than 30 years old	1.17(.58–2.37)
								paternal consanguineous marriage	2.58(.76–8.71)
Nasri et al. 2016 [54]	Case-control	Tunisia	Newborn and mothers	150	75	75	Medium	maternal age greater than 30 years old	1(.53–1.86)
								folic acid supplementation	1.18(.61–2.31)
								previous history of stillbirth	2.09(.82–5.28)
Gadefaw et al. 2016 [38]	Cross-sectional	Ethiopia	Newborn and mothers	333	111	222	Medium	maternal age greater than 30 years old	2(.73–5.47)
								previous history of stillbirth	.49(.10–2.35)
								sex of newborn	.67(.47–1.04)
Birhane et al. 2018 [42]	Case-control	Ethiopia	Newborn and mothers	617	205	412	Low	maternal age greater than 30 years old	2.46 (1.33–4.53)
								maternal alcohol consumption	10.28 (1.19–88.50)
								folic acid supplementation	2.15 (1.02–4.54)
								exposure to pesticide	5 (0.150–166.60)
								exposure to radiation	5 (0.150–166.60)
Filmawit et al. 2018 [40]	Case-control	Ethiopia	Newborn and mothers	180	60	120	Medium	maternal alcohol consumption	.56(.21–1.46)
								previous history of stillbirth	.66(.067–6.42)
								exposure to pesticide	2.02(.978–4.17)
								exposure to radiation	1.34(.217–8.27)
								sex of newborn	1.71(.914–3.2)
Bourouba et al. 2018 [18]	Case-control	Algeria	Newborn and mothers	133	48	85	Medium	folic acid supplementation	4.15 (0.89–19.25)
								paternal consanguineous marriage	0.92 (0.42–1.97)
Wolderufael et al. 2018 [37]	Case-control	Ethiopia	Newborn and mothers	647	205	412	Low	folic acid supplementation	.48(.23–1.02)
Atlaw et al. 2018 [39]	Case-control	Ethiopia	Newborn and mothers	462	42	420	Medium	maternal alcohol consumption	.79 (.32–1.98)
								folic acid supplementation	.09 (.031–.285)
								previous history of stillbirth	1.41 (.42–4.75)
								exposure to radiation	.44 (.09–2.08)
								paternal consanguineous marriage	4.9 (1.49–16.17)
								exposure to pesticide	.19 (.02–2.21
								sex of newborn	.72 (.381–1.37)

### Prevalence of neural tube defects (NTDs) in Africa

**Fig. 2:**
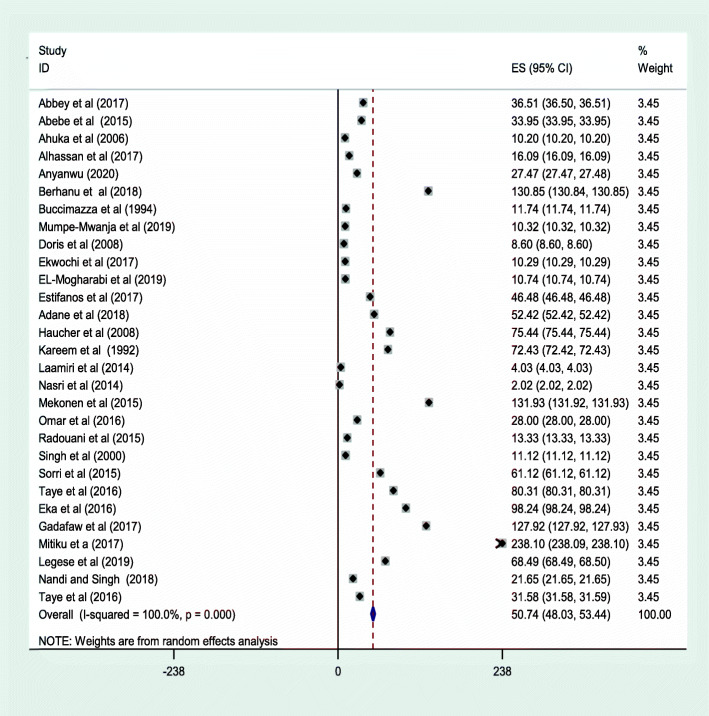
Meta-analysis, the prevalence of neural tube defects per 10,000 births in Africa

**Fig. 3:**
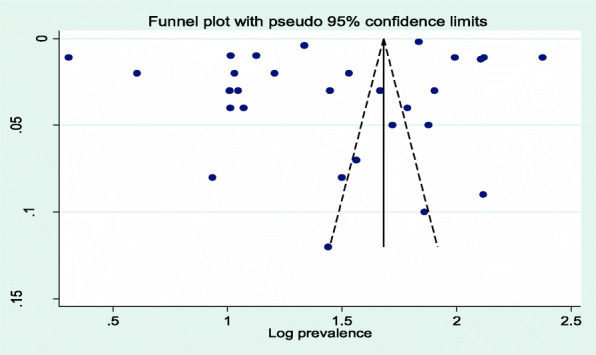
Funnel plot showing publication bias status of studies included for the meta-analysis on prevalence of neural tube defects in Africa

Twenty-nine articles were included in the meta-analysis to estimate the prevalence of NTD in Africa. A total of 6,113,208 newborns were included in the analysis. The included studies reported a sample size, ranging from the minimum of 84 participants in the Ethiopian study [27] to the maximum of 3,803,889 in the Tunisian study [51]. The result of random-effect meta-analysis estimated the pooled prevalence of NTDs in Africa was 50.71 per 10,000 births (95% CI: 48.03, 53.44; I^2^ = 100%, *p* < 0.001) (Fig. 2**).** There was significant heterogeneity among the included studies (I^2^ = 100%, *p*-value < 0.001). Begg’s test showed that there is no statistically significant publication bias with *p*-value = 0.743 (Fig. 3).

### Sub-group analysis of the prevalence of NTDs

**Fig. 4:**
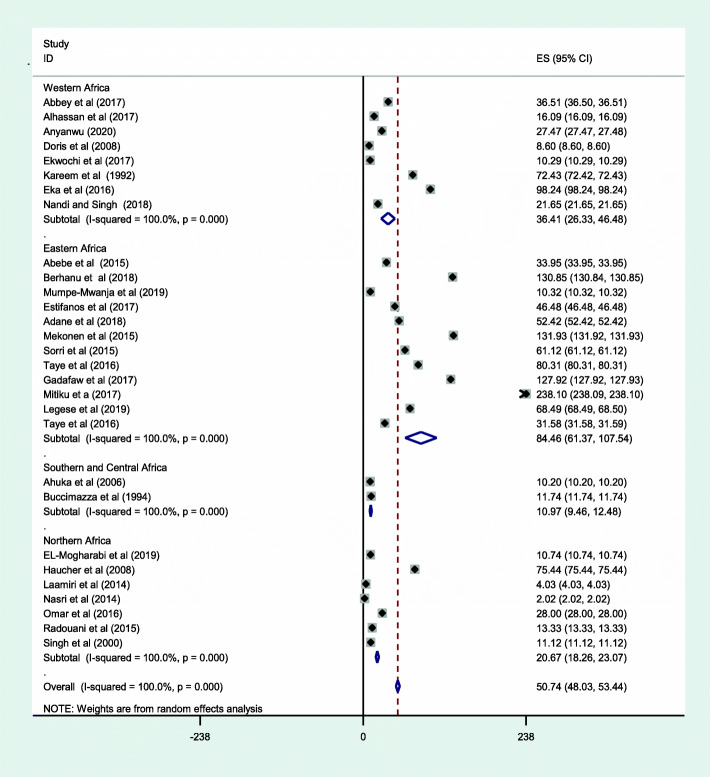
Sub-group analysis by the regions of African countries, the prevalence of neural tube defects per 10,000 births in Africa

The sub-group analysis by sub-region showed that the prevalence of NTD was highest in the East African sub-region, 84.48 cases of NTDs per 10,000 births (95% CI: 61.37, 107.54), and the lowest was in Central Africa, 10.20 per 10,000 births (95% CI: 10.20, 10.20) (Fig. 4**).**

### Prevalence of NTDs by sub-types in Africa

**Fig. 5:**
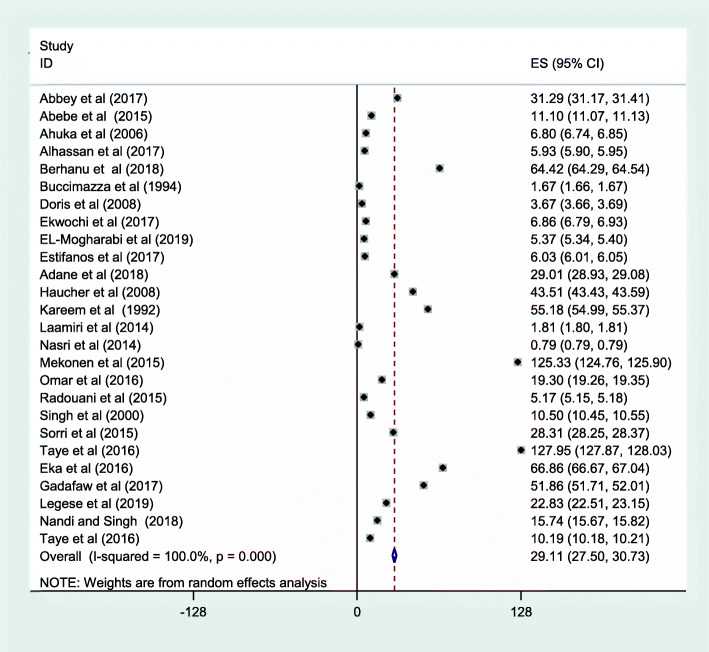
Meta-analysis, the prevalence of spina bifida per 10,000 births in Africa

**Fig. 6:**
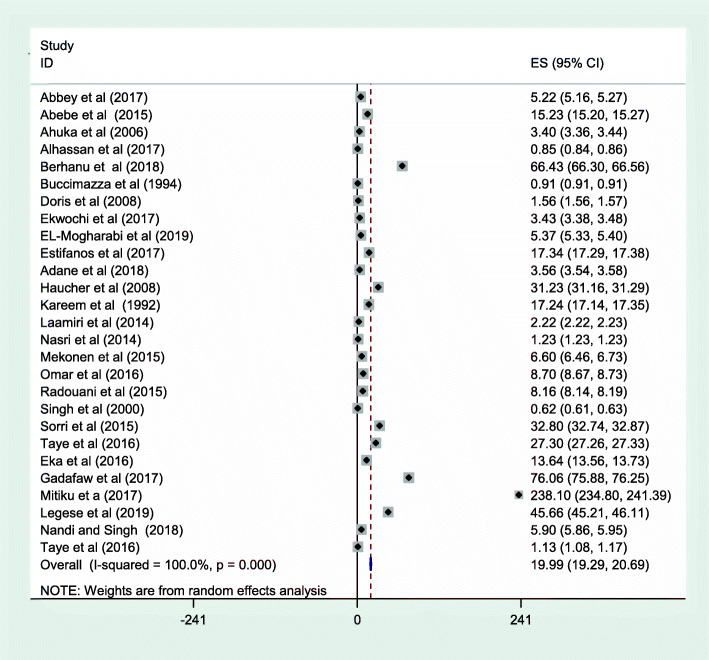
Meta-analysis, the prevalence of anencephaly per 10,000 births in Africa

The meta-analysis was also conducted by sub-type of neural tube defect. In this sub-group, the prevalence of spina bifida (cystica and occulta) was 29.67 cases per 10,000 births (Fig. 5), and anencephaly was 19.11 cases per 10,000 births (Fig. 6).

#### Determinants of neural tube defects (NTDs)

The determinant factors included in this analysis were maternal age, maternal folic acid supplementation before pregnancy and within the first month of pregnancy, paternal consanguineous marriage, previous history of stillbirth, exposure to a pesticide, exposure to radiation, history of alcohol consumption, and sex of the newborn. A separate analysis was conducted for each variable.

### Folic acid and neural tube defects

**Fig. 7:**
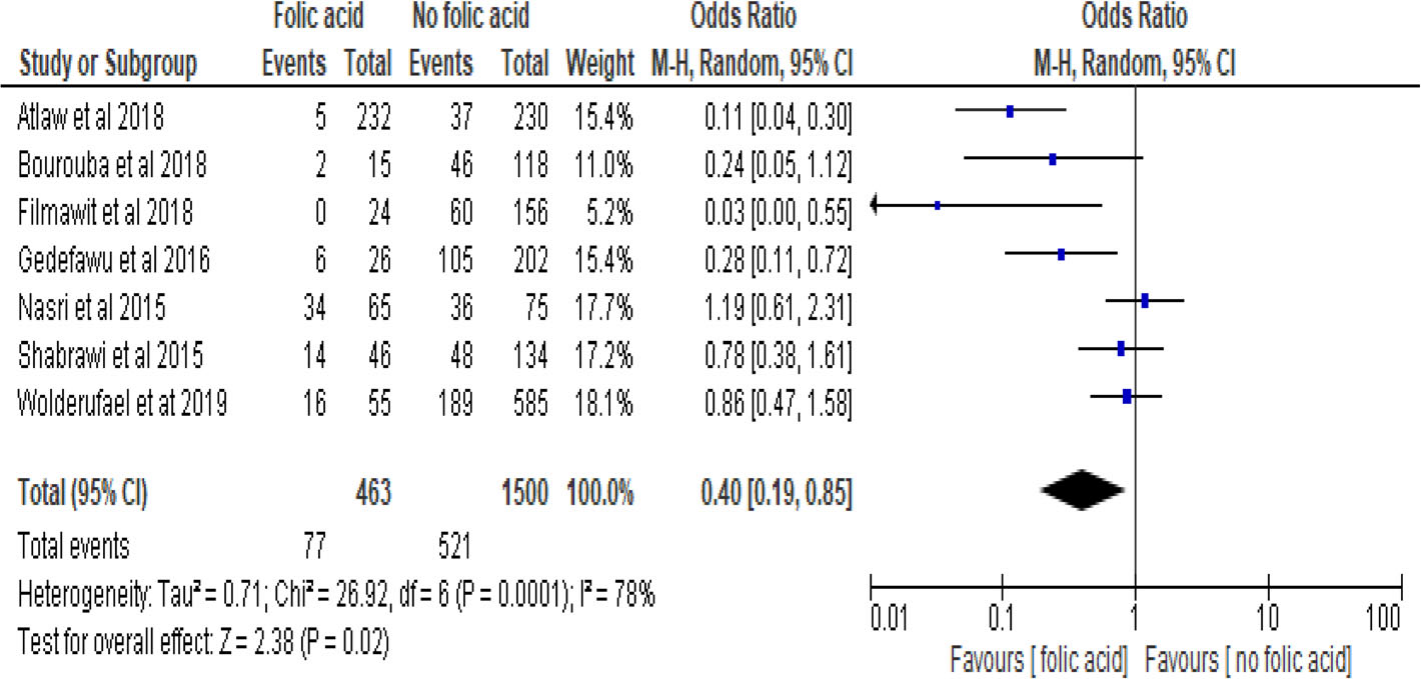
Forest plot showing association between folic acid supplements and neural tube defects in Africa

Six studies [18, 37–40, 51, 52] examined the association between folic acid supplementations before and within the first months of pregnancy. The pooled odds ratio indicated that women who have taken folic acid supplements before pregnancy and within the first month of pregnancy were 60% less likely to have newborns with neural tube defects (POR, 95% CI:0.4 (0.19–0.85)). The studies showed high heterogeneity (I^2^ = 78.0% and *p* < 0.001). Hence a random effect model was employed for final analysis (Fig. 7**)**.

### Paternal consanguineous marriage and neural tube defects

**Fig. 8:**
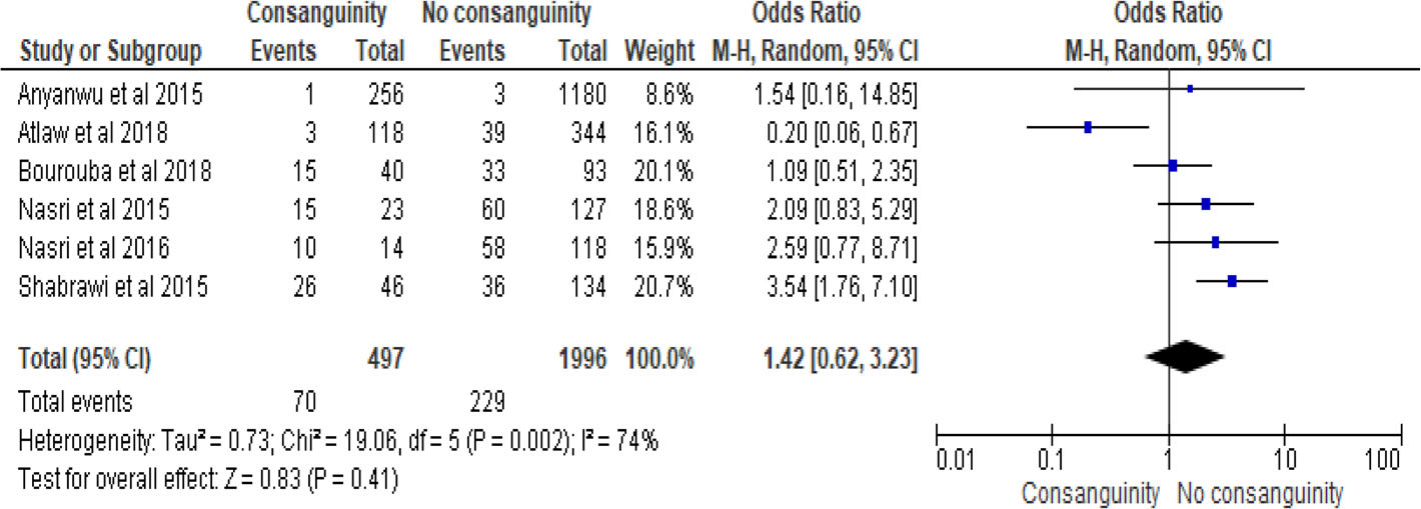
Forest plot showing association of consanguineous marriage and neural tube defects in Africa

The association of paternal consanguineous marriage and neural tube defect was examined based on the findings from six studies [17, 18, 39, 52, 54, 55]. The pooled odds ratio indicated that neural tube defects do not differ among parents with and without consanguineous marriages (POR, 95% CI: 1.42 (.62–3.23)). The studies showed moderate heterogeneity (I^2^ = 74.0%, *p* < 0.001). Hence a random effect model was considered for the final analysis (Fig. 8).

#### Exposure to pesticide and neural tube defects

**Fig. 9:**
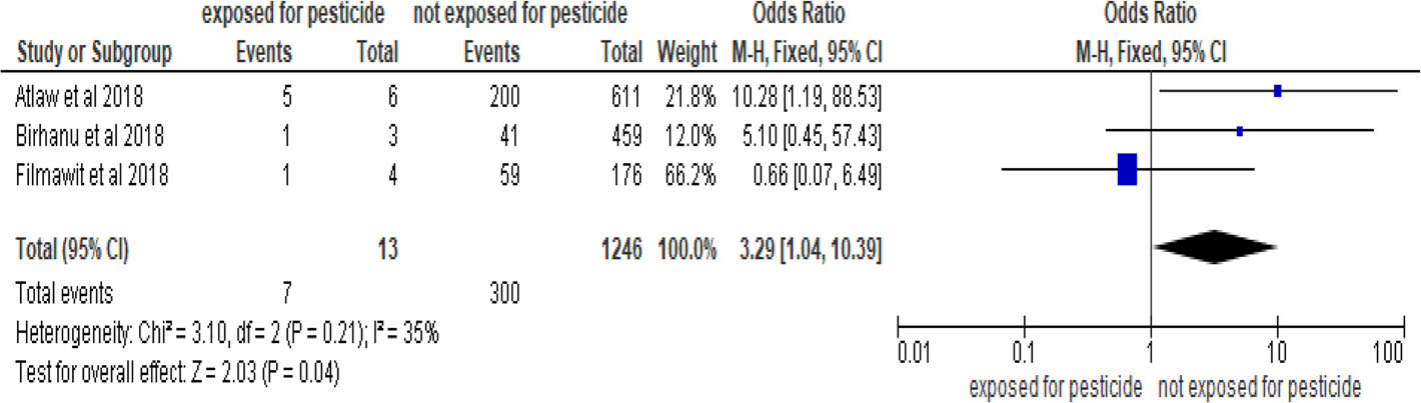
Forest plot showing association between pesticide exposure and neural tube defects in Africa

This meta-analysis was employed on three studies [32, 33, 53], and pooled odds ratio was examined. It revealed that women exposed to the pesticide were three times more likely to have newborns with neural tube defects than women who were not exposed to pesticide during pregnancy and within 1 year before pregnancy (POR, 95% CI: 3.29 (1.04–10.39)). The studies showed low heterogeneity (I^2^ = 35.0% and *p* = 0.21) (Fig. 9). Hence a fixed-effect model was considered to do the final analysis. A funnel plot was symmetrical.

#### Maternal age and neural tube defects

The association of maternal age and neural tube defect was examined based on the findings from five studies [38, 40, 42, 51, 54]. The pooled odds ratio indicated that the odds of neural tube defect are 1.5 times higher among women with an age group greater than 30 years during pregnancy (POR, 95% CI: 1.47(1.16–1.87)). The heterogeneity test revealed mild heterogeneity (I^2^ = 86%, *p* = < 0.001), and therefore, a random effect model was used in the final analysis (Table 3).

**Table 3:** Showing relationship of different factors with the neural tube defects in Africa

Variables	Pooled odds ratio with 95% CI	I^**2**^%	***p***-value
x-ray radiation exposure	2.34 (1.27–4.31)	0	*P* < 0.001
Alcohol consumption	1.3 (0.41–4.48)	67	0.05
History of stillbirth	3.35 (1.99–5.65)	86	0.001
Sex of newborn	0.86 (0.60–1.23)	43	0.17
Age	1.47 (1.16–1.87)	85	*p* < 0.001

#### Maternal exposure to radiation and neural tube defects

Three studies examined the association between maternal exposure to pesticides during pregnancy [39, 40, 42]. The pooled estimate showed that women exposed to x-ray radiation were two times more likely to have newborns with neural tube defects than women who were never exposed to x-ray radiation (POR, 95% CI: 2.34 (1.27–4.31)). The heterogeneity test revealed low heterogeneity (I^2^ = 0%, *p* = 0.1), and therefore a fixed model was assumed in the analysis (Table 3).

#### Maternal alcohol consumption and neural tube defects

Pooled results of three studies [39, 40, 42] showed that neural tube defect occurrence does not differ among women who consume alcohol and who do not consume alcohol during their pregnancy and 1 year before pregnancy (POR, 95% CI: 1.3 (0.41–4.48)). The heterogeneity test revealed mild heterogeneity (I^2^ = 67%, *p* = 0.05), and therefore a random model was assumed in the analysis (Table 3).

#### Previous stillbirth and neural tube defects

Four studies [38–40, 42] have examined the association of the previous stillbirth and neural tube defects. The pooled odds revealed that women who have a previous history of stillbirth were three times more likely to have newborns with neural tube defects than women who have no previous stillbirth [POR, 95% CI: 3.35 (1.99–5.65)]. The heterogeneity test revealed high heterogeneity (I^2^ = 86%, *p* = < 0.001) (Table 3**),** and therefore, a random model was assumed in the analysis.

#### Sex of newborn and neural tube defect

Three studies [38–40] included in the meta-analysis have revealed that there was no difference among male and female newborns on the occurrence of neural tube defects (POR, 95% CI: 0.86 (0.60–1.23)). The heterogeneity test revealed mild heterogeneity (I^2^ = 43%, *p* = < 0.17) (Table 3**),** and therefore, a fixed-effect model was used in the analysis.

## Discussion

This systematic review and meta-analysis were conducted to estimate the prevalence and determinant factors of neural tube defects (NTDs) in Africa. The pooled prevalence of NTDs in Africa was 50.74 per 10,000 births. This prevalence is higher than a previous review conducted in low and middle-income countries (LMICs) in 2015, which reported 11.7 per 10,000 births [3]. The previous review included only two studies from the African region, which might underestimate the overall prevalence in the region. Besides, our finding is much higher than reported by a review conducted in India [56] which might be attributable to the geographical and sociodemographic variation of study participants.

The subgroup analysis has shown a higher prevalence of NTD in East African countries with 84.84 cases per 10,000 births which may be due to higher serum folic acid deficiency among reproductive-age women in Eastern Africa countries [57]. A limited number of Eastern Africa countries have the mandatory folic acid fortification of staple food in the African region [58]. A lower prevalence of NTDs was observed in Central and Southern Africa. In this region, the studies included those from Cameroon and South Africa, which have mandatory folic acid fortification that might affect the prevalence of NTDs [59].

This study also tried to identify the most common avoidable factors of neural tube defects in Africa. The present meta-analysis indicated that women who have taken folic acid supplements before pregnancy and/or within the first month of pregnancy were less likely to have newborns with neural tube defects. These findings are in line with a review that showed evidence of decreased risk of NTDs occurring among women who have folic acid supplements [60]. A review study conducted in 2017 by the US task force has revealed the protective effect of folic acid on neural tube defects [61]. The folic acid increases neural tube closure and central nervous system development as it has a role in nucleotide synthesis, and folate also engages acute signaling in neurons [60, 62–64].

The findings of this pooled odds ratio indicated that NTD does not differ among parents with and without consanguineous marriages. This finding contradicts with a review conducted in 2010 that has revealed consanguinity as a significant risk of NTDs [65]. The consanguineous marriages will increase autosomal recessive and dominant ending up in neural tube defects [66, 67]. The difference may be due to the presence of limited primary studies investigating the association of NTDs and consanguinity in this review and meta-analysis.

The pooled odds indicated that the odd neural tube defect is 1.5 times higher among women with an age group of greater than 30 years during pregnancy. This observed difference can be explained by the fact that an increase in age will increase the risk of aneuploidy [68, 69]. Besides, a gradual loss of meiotic cohesion during meiosis in oocytes contributes to non-disjunction events leading to a high incidence of aneuploidy [70]. Aneuploidy, in turn, will result in neural tube defects [71].

Women with a history of agricultural pesticide exposure during the first 3 months of pregnancy were three times more likely to give birth to newborns with neural tube defects than those with no history of exposure to a pesticide. This finding is supported by a study conducted in America, where mothers having pesticide exposure were 3 times more likely to have newborns with NTDs [72]. This high risk might be explained by the effect of pesticides on nucleotide synthesis [73, 74]. Pesticide exposure results in impaired development if it occurs in early pregnancy [75, 76]. Further, organochlorine pesticide increases the risk of NTDs [77], and this pesticide is a commonly used agricultural pesticide in Africa [78]. Since most of the African population are farmers, they are subjected to frequent exposure to agricultural pesticides [77].

The present meta-analysis identified that women with a history of exposure to x-ray radiation during the first 3 months of pregnancy were two times higher odds of having a newborn with a neural tube defect than women with no history of exposure. This observation might be due to x-ray ionization that may result in genetic mutation, which in turn affects DNA methylation, thereby ending up in NTDs [79]. A review conducted in 2015 concluded that low-dose radiation exposure could damage the DNA [80]. Further, radiation will induce neuronal apoptosis even at a low dose was evidenced in developing neocortex [81], which results in apoptotic mutation that causes failure of neural tube closure [81, 82].

Women who have a previous history of stillbirth were three times more likely to have newborns with neural tube defects than women who have no previous stillbirth. This can be explained by the fact that women who have a previous history of NTD have the risk of recurrences in a subsequent pregnancy [83, 84]. The common cause for recurrence of NTDs was low serum folate and chromosomal abnormalities [85]. Further, Neural tube defect was shown to have 25 and 50% recurrence among autosomal recessive and dominant, respectively [86].

Generally, the burden of NTD is higher in Africa when compared to other regions, and identified factors are avoidable by folic acid fortification and health education provision on the effects of pesticides and ionizing radiation.

### Limitations

There are limited original studies conducted in central and southern regions of Africa which may affect the overall burden of NTDs in Africa. There were few primary studies conducted on factors associated with NTDs, which may affect the representation of Africa. The original studies included in this meta-analysis did not report the dose, duration, and timing of exposure to x-ray radiation that may affect the strength of association between NTDs and x-ray radiation.

## Conclusion

This meta-analysis and systematic review identified that there is a high burden of NTDs in Africa. About five in thousand newborns are affected in Africa, while nine per thousand newborns are affected in Eastern Africa. Before and during early pregnancy, folic acid supplementation was identified as protective factors, while the previous history of stillbirth, exposure to a pesticide, and x-ray radiation were factors associated with NTDs in Africa.

Most of the factors identified in this study were avoidable; therefore, folic acid fortification of staple foods and considering folic acid supplementation of reproductive-age women may significantly reduce a high burden of NTDs in Africa. Further, avoiding pesticide and x-ray exposures during the first trimester of pregnancy may also contribute to the reduction of NTDs occurrence. In addition, we recommend original studies to be conducted in the Central and Southern African regions.

## Supplementary Information

**Additional file 1.** PRISMA checklist.

**Additional file 2.** Search stratagy and terms used to find articles from databeses.

**Additional file 3.** Risk of bias assesement tool for cross-sectional studies.

**Additional file 4.** Risk of bias assesement tool for case-control studies.

**Additional file 5.** Data extraction tool for magnitude of NTDs.

**Additional file 6.** Data extraction tool for associated factors with NTDs.

## Data Availability

The part of the data analyzed during this study is included in this manuscript. Other data will be available from the corresponding author upon a reasonable request.

## Abbreviations

CI: Confidence Interval
JBI: Joanna Briggs Institute
PRISMA: Preferred Reporting System for Meta-Analysis and Systematic Review
NTDs: Neural Tube Defects
POR: Pooled Odds Ratio
WHO: World Health Organization
