# Supplementary material for: Magnitude and determinants of neural tube defect in Africa: a systematic review and meta-analysis
Source: BMC Pregnancy Childbirth. 2021 Jun 14;21:426. doi: 10.1186/s12884-021-03848-9 (PMC8204447; doi:10.1186/s12884-021-03848-9)
Supplement: Supplementary file 2 — Additional file 2. Search stratagy and terms used to find articles from databeses. [file 12884_2021_3848_MOESM2_ESM.docx]

Example of search on PubMed

Search: **(((neural) AND (tube)) AND (defect)) AND (africa)**

("neural"[All Fields] OR "neuralization"[All Fields] OR "neuralize"[All Fields] OR "neuralized"[All Fields] OR "neuralizes"[All Fields] OR "neuralizing"[All Fields] OR "neurally"[All Fields]) AND "tube"[All Fields] AND ("abnormalities"[MeSH Subheading] OR "abnormalities"[All Fields] OR "defects"[All Fields] OR "defect"[All Fields] OR "defect s"[All Fields] OR "defected"[All Fields] OR "defective"[All Fields] OR "defectively"[All Fields] OR "defectives"[All Fields]) AND ("africa"[MeSH Terms] OR "africa"[All Fields] OR "africa s"[All Fields] OR "africas"[All Fields])

**Translations**

**neural:** "neural"[All Fields] OR "neuralization"[All Fields] OR "neuralize"[All Fields] OR "neuralized"[All Fields] OR "neuralizes"[All Fields] OR "neuralizing"[All Fields] OR "neurally"[All Fields]

**defect:** "abnormalities"[Subheading] OR "abnormalities"[All Fields] OR "defects"[All Fields] OR "defect"[All Fields] OR "defect's"[All Fields] OR "defected"[All Fields] OR "defective"[All Fields] OR "defectively"[All Fields] OR "defectives"[All Fields]

**africa:** "africa"[MeSH Terms] OR "africa"[All Fields] OR "africa's"[All Fields] OR "africas"[All Fields]

Search: **((((neural) AND (tube)) AND (defect)) AND (africa)) AND ((((("associated factor"[All Fields] OR "determinant factor"[All Fields]) OR "risk factor"[All Fields]) OR "preventive factor"[All Fields]) AND ("africa"[MeSH Terms] OR "africa"[All Fields])))**

("neural"[All Fields] OR "neuralization"[All Fields] OR "neuralize"[All Fields] OR "neuralized"[All Fields] OR "neuralizes"[All Fields] OR "neuralizing"[All Fields] OR "neurally"[All Fields]) AND "tube"[All Fields] AND ("abnormalities"[MeSH Subheading] OR "abnormalities"[All Fields] OR "defects"[All Fields] OR "defect"[All Fields] OR "defect s"[All Fields] OR "defected"[All Fields] OR "defective"[All Fields] OR "defectively"[All Fields] OR "defectives"[All Fields]) AND ("africa"[MeSH Terms] OR "africa"[All Fields] OR "africa s"[All Fields] OR "africas"[All Fields]) AND (("associated factor"[All Fields] OR "determinant factor"[All Fields] OR "risk factor"[All Fields] OR "preventive factor"[All Fields]) AND ("africa"[MeSH Terms] OR "africa"[All Fields]))

**Translations**

**neural:** "neural"[All Fields] OR "neuralization"[All Fields] OR "neuralize"[All Fields] OR "neuralized"[All Fields] OR "neuralizes"[All Fields] OR "neuralizing"[All Fields] OR "neurally"[All Fields]

**defect:** "abnormalities"[Subheading] OR "abnormalities"[All Fields] OR "defects"[All Fields] OR "defect"[All Fields] OR "defect's"[All Fields] OR "defected"[All Fields] OR "defective"[All Fields] OR "defectively"[All Fields] OR "defectives"[All Fields]

**africa:** "africa"[MeSH Terms] OR "africa"[All Fields] OR "africa's"[All Fields] OR "africas"[All Fields]
